# Clinician Identification of Birth Asphyxia Using Intrapartum Cardiotocography Among Neonates With and Without Encephalopathy in New Zealand

**DOI:** 10.1001/jamanetworkopen.2019.21363

**Published:** 2020-02-19

**Authors:** Cynthia M. Farquhar, Sarah Armstrong, Vicki Masson, John M. D. Thompson, Lynn Sadler

**Affiliations:** 1 Department of Obstetrics and Gynaecology, University of Auckland, Auckland, New Zealand; 2 Auckland District Health Board, Auckland, New Zealand

## Abstract

**Importance:**

Despite improvements in antenatal care and increasing cesarean delivery rates, birth asphyxia leading to neonatal encephalopathy (NE) continues to contribute to neonatal death and long-term neurodevelopmental disability. Cardiotocography (CTG) has been used in labor for several decades to detect a stressed fetus so that delivery can be expedited and NE avoided.

**Objective:**

To investigate whether experienced clinicians can detect and respond to abnormal readings from CTGs during the penultimate hour before birth in infants with moderate to severe NE but no acute peripartum event.

**Design, Setting, and Participants:**

This case-control study included 10 practicing obstetricians and midwives at maternity hospitals in New Zealand. Participants, who were masked to the perinatal outcome, were asked to assess CTG tracings from 35 neonates with NE and evidence of birth hypoxia (ie, cases) and 105 neonates without NE or birth hypoxia (ie, controls), all of whom were born in 2010 to 2011. Data analysis was conducted from May to December 2017.

**Exposures:**

Brief clinical details and 1 hour of CTG tracings from the penultimate hour before birth were provided for each baby. Clinicians assessed the CTG tracings and recommended a plan.

**Main Outcomes and Measures:**

Intra-assessor and interassessor agreement on CTG findings and action plans as well as sensitivity (ie, detection of NE) and specificity (ie, ruling out those without NE) for the assessment of abnormal CTG readings leading to immediate action (ie, fetal blood sample or immediate delivery) were reported.

**Results:**

A total of 35 infants (mean [SD] gestational age, 40 [1.4] weeks; 16 [45.7%] cesarean deliveries) were designated cases, and 105 infants (mean [SD] gestational age, 39.4 [1.2] weeks; 22 [21.0%] cesarean deliveries) were designated controls. No infants had congenital anomalies. The mean (range) sensitivity for detection of abnormal CTG results and for recommending immediate action for all assessors was 75% (63%-91%) and 41% (23%-57%), respectively, with a mean (range) specificity of 67% (53%-77%) and 87% (65%-99%), respectively. A sensitivity analysis including only assessors with 80% or more interassessor agreement only differed from the main analysis by 6% or less (mean [range] sensitivity for detection, 76% [63%-91%]; sensitivity for action plan, 36% [25%-49%]; specificity for detection, 71% [53%-77%]; and specificity for action plan, 93% [88%-99%]).

**Conclusions and Relevance:**

Experienced clinicians detected 3 of 4 infants who were subsequently diagnosed with NE. Action to expedite delivery was recommended for more than 40% of infants with NE. These results indicate that CTG does not identify all infants at risk of NE, and that there is a need for further investment in new approaches to fetal surveillance in labor.

## Key Points

**Question:**

Can experienced clinicians detect and manage abnormal cardiotocograph readings during the penultimate hour before birth among infants with moderate to severe neonatal encephalopathy but no acute peripartum event?

**Findings:**

In this case-control study of 35 infants with neonatal encephalopathy and 105 without, experienced obstetricians and midwives were able to detect 3 of 4 neonates who were subsequently diagnosed with encephalopathy using cardiotocography. Immediate action was recommended for more than 40% of infants with encephalopathy.

**Meaning:**

The findings of this study indicate that further investment in new approaches to intrapartum fetal surveillance is needed.

## Teaser

This case-control study investigates whether experienced clinicians can detect abnormal readings from cardiotocography during the penultimate hour before birth among infants in New Zealand with moderate to severe neonatal encephalopathy but no acute peripartum event.

## Introduction

Despite improvements in antenatal care and increasing cesarean delivery rates, birth asphyxia leading to neonatal encephalopathy (NE) continues to contribute to rates of stillbirth, neonatal death, and long-term neurodevelopmental disability.^1,2,3,4,5^ Neonatal encephalopathy is defined as disordered neonatal brain function within the first week of life in term infants (ie, 37 weeks or older).^6^ Approximately 30% of cerebral palsy occurs in infants diagnosed with NE, and NE is also a leading cause of medical litigation.^6,7,8^ Hypoxic peripartum injury is identified in more than 50% of instances of NE in New Zealand.^8,9^ In 2014, the American Academy of Pediatricians and the American College of Obstetricians and Gynecologists called for in-depth analysis of cases to identify contributing factors among babies born with asphyxia.^8^

Cardiotocography (CTG) in labor has been used for more than 3 decades to detect a stressed fetus so that delivery can be expedited in an attempt to reduce asphyxia leading to NE.^10,11,12,13^ Continuous CTG monitoring in labor has been shown to halve neonatal seizures.^13^ It is a controversial area, and it is generally agreed that more research is required.^6,14,15,16^ In New Zealand, CTG is used liberally; however, a small number of babies (ie, 1.27 per 1000 term births in 2010-2011) are still diagnosed with NE within the first week of life.^9,10,17,18^ This could occur because the CTG readings are normal (poor sensitivity), the CTG readings are abnormal but are interpreted as normal (detection error), the CTG readings are interpreted as abnormal but no action is taken (management error), it is too late to prevent NE, or NE is due to a cause other than peripartum asphyxia.

In 2013, an independent multidisciplinary team of the Perinatal and Maternal Mortality Review Committee (PMMRC) of New Zealand extensively reviewed the care of infants born from 2010 to 2011 and subsequently diagnosed with NE whose mothers labored without an acute peripartum event.^9,10,17,19^ The review of cases identified that detection and management errors were common and contributed to NE. These concerns included the interpretation of CTG results and the actions taken to expedite delivery. The review noted that review teams could not fairly assess CTGs retrospectively, given that they had full knowledge of the outcomes.

The objective of the current study was to determine whether experienced clinicians could detect abnormal CTG readings taken during the penultimate hour before delivery in babies diagnosed with moderate to severe NE and recommend an appropriate action plan. Clinicians were masked to the outcome, and CTGs from neonates with NE were nested randomly with CTGs from infants with normal lactate levels.

## Methods

Access to clinical material for the cases included in this study was enabled by the New Zealand Public Health and Disability Act legislation that covers PMMRC activities. The Health and Disability Ethics Committee of the Ministry of Health approved the study in March 2014, and additional approval was given by the Counties Manukau District Health Board Health Research Committee for the use of the controls, who were accessed from a study with ethical approval granted by the Health and Disability Ethics Committee in September 2014. Informed consent was not required for cases under the New Zealand Public Health and Disability Act, but informed consent was obtained for control group data. The study followed the Strengthening the Reporting of Observational Studies in Epidemiology (STROBE) reporting guideline.

The Neonatal Encephalopathy Working Group of the PMMRC has prospectively collected data on cases of moderate and severe NE among term neonates in New Zealand since 2010.^17,19^ The PMMRC defines NE as a clinically defined syndrome of disturbed neurological function within the first week of life in term infants (ie, 37 weeks or older), manifested by difficulty in initiating and maintaining respiration, depression of tone and reflexes, subnormal level of consciousness, and, often, seizures.^17^

### Inclusion Criteria

Of the 149 NE cases reported in NZ from 2010 to 2011, 83 (55.7%) had no evidence of an acute peripartum event during labor and were reviewed in detail. Of these, 35 (42.2%) had at least 1 hour of CTG recording of adequate quality from 2 hours before birth^9^ (Figure). The cases were infants born in New Zealand from 2010 to 2011 at a gestational age of 37 weeks or older without congenital anomalies who met the PMMRC definition of moderate to severe NE and showed evidence of hypoxia at birth. No evidence of an antenatal cause of NE or of an acute peripartum event that would explain the diagnosis of NE was found.^9^ Multiple pregnancies were included. The blood gases and 1-minute Apgar scores for each infant are presented in eTable 1 in the Supplement. All infants were delivered after labor and had a CTG started at least 2 hours before delivery for at least 1 hour. Overall, 26 of 35 infants (74.3%) received therapeutic hypothermia. The cases were all those included in the published review who met the inclusion criteria for this study and who had a readable CTG.^9,10^

**Figure.**
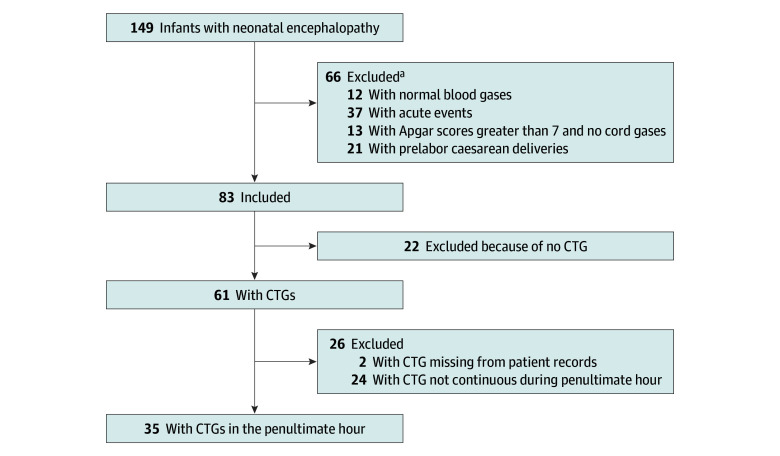
Flow Diagram of Included Cases, Identified From a National Cohort of Neonates With Encephalopathy CTG indicates cardiotocograph. ^a^Infants could have more than 1 reason for exclusion.

The controls were selected at random from a cohort of infants born in 2010 to 2011 at 1 tertiary hospital in New Zealand for an unrelated study of cord lactate.^20^ They had a gestational age of 37 weeks or older, with Apgar scores of 9 or 10 at 1 and 5 minutes, cord lactate of 4.8 mmol/L or less,^20^ at least 1 hour of CTG recordings from at least 2 hours before delivery, no evidence of NE, no congenital anomalies, and no admission to a neonatal intensive care unit.

### Clinical Assessors

Practicing midwives and obstetricians were invited through clinical networks. Clinicians were required to have completed the Royal Australian and New Zealand College of Obstetricians and Gynaecologists (RANZCOG) fetal surveillance education program^11^ within the previous 2 years and to be practicing on the labor ward. The panel consisted of 5 practicing midwives and 5 obstetricians. All clinicians were currently working on a labor ward with a median (range) of 16 (4-26) years of experience. Assessors were informed that the study was a quality improvement project, were not informed that cases were diagnosed with NE, and were not informed of perinatal outcome.

### Online Survey

An online survey was constructed of CTG recordings for cases and controls for the penultimate hour before delivery. Participants were provided a brief clinical history, including liquor description, labor induction, oxytocin use, strength and frequency of contractions, vaginal and abdominal examination findings, maternal observations, and pain relief. Cases were placed randomly among controls. The assessors were asked to complete the RANZCOG CTG assessment tool, including baseline rate, variability, presence of accelerations and decelerations; give a summary assessment of the CTG reading (ie, normal, suspicious, or abnormal); and provide a plan for subsequent clinical care (Table 1; eTable 2 in the Supplement).^7^

**Table 1.** Royal Australian and New Zealand College of Obstetricians and Gynaecologists Cardiotocography Assessment Tool

Finding	Normal	Suspicious	Abnormal
Baseline rate, beats/min	110-160	100-109	<100 or >160
Variability	≥5 Beats/min	<5 Beats/min for 40-90 min	<5 Beats/min for ≥90 min
Accelerations	Present	None	None
Decelerations	None	Early decelerations or single variable deceleration up to 3 min	Repeated variable or late decelerations or prolonged deceleration lasting >3 min
Overall opinion	All features normal	1 Suspicious feature	≥2 Suspicious features or ≥1 abnormal features

There was no time limit for completing the online reviews. A year after the initial online survey was sent to assessors, the same assessors were asked to repeat assessments of the first 30 original cases and controls.

### Data Collection and Outcomes

Maternal age, parity, onset of labor (ie, spontaneous or induced), oxytocin use, temperature in labor, dilatation at last vaginal examination, epidural use and timing of epidural top up, length of labor, mode of delivery, neonatal outcome, and gestational age were extracted from clinical notes for both cases and controls. We reported 3 primary outcomes, as follows: the proportion of cases in which assessors rated a CTG reading as abnormal (ie, sensitivity for detection of hypoxia); the proportion of cases in which assessors planned either fetal blood sampling or immediate delivery by cesarean or instrumental delivery, dependent on their clinical findings (ie, sensitivity for appropriate management of hypoxia); and the proportion of controls in which assessors rated a CTG reading as normal and made no plan for immediate action (ie, specificity).

### Power Calculation

The case sample included all eligible infants born in New Zealand in 2010 to 2011 who fulfilled the inclusion criteria. Power calculations assuming variable rates of immediate action in response to abnormal CTG readings among the controls and cases were undertaken. This helped to inform the number of controls included in the study. The study had 80% power at a significance level of .05 to detect a difference in rates of immediate action in response to abnormal CTG readings, assuming action rates of 1% and 15%, 5% and 25%, and 10% and 35% in controls and cases, respectively.

### Statistical Analysis

There was a priori concern to ensure the expertise of the assessors was optimized. Therefore, interassessor reliability was assessed as a measure of assessor quality. A sensitivity analysis was conducted excluding those whose interassessor agreement with any other assessor on the action plan was less than 80%. The assessors included in the sensitivity analysis (ie, those with ≥80% agreement) were termed *reliable assessors*. The rationale for this exclusion was to optimally assess and report on the potential for CTGs to reduce NE in infants. Statistical comparisons of proportions and continuous variables were performed using χ^2^ tests and *t* tests, with *P* < .05 denoting statistical significance. All tests were 2-tailed. Analyses were conducted with SAS version 9.4 (SAS Institute).

## Results

A total of 35 infants (mean [SD] gestational age, 40 [1.4] weeks; 16 [45.7%] cesarean deliveries) were designated cases, and 105 infants (mean [SD] gestational age, 39.4 [1.2] weeks; 22 [21.0%] cesarean deliveries) were designated controls. All infants were delivered at term without congenital anomalies. Infants in the control group had normal lactate levels and Apgar scores of 9 and 10. The demographic and clinical characteristics of all mothers and neonates are reported in Table 2.

**Table 2.** Summary of Maternal and Intrapartum Characteristics and Blood Gases

Characteristic	No. (%)		*P* Value
	Cases (n = 35)	Controls (n = 105)	
Maternal age, mean (SD), y	28.9 (5.8)	27.6 (5.3)	.21
First birth	10 (28.6)	45 (42.9)	.10
Gestational age, mean (SD), wk	40 (1.4)	39.4 (1.2)	.02
Induction of labor	13 (37.1)	42 (40.0)	.61
Multiple pregnancy	1 (2.9)	2 (1.9)	.74
VBAC	3 (8.6)	11 (10.5)	.74
Mode of birth			
Vaginal breech delivery	1 (2.9)	0	<.001
Cesarean delivery	16 (45.7)	22 (22.0)	
Operative vaginal delivery	10 (28.6)	4 (3.8)	
Spontaneous vaginal delivery	8 (22.9)	63 (60.0)	
Fever in labor >38 °C	1 (3.1)	3 (2.9)	>.99
Meconium	13 (37.1)	26 (24.8)	.16
Preeclampsia	1 (2.9)	3 (2.9)	.97
BMI ≥30	8 (22.9)	42 (40.0)	.02
Smoker	10 (28.6)	17 (16.2)	.13
Gestational or preexisting diabetes	2 (5.7)	14 (13.3)	.22
Dilatation in penultimate hour, mean (SD), cm	6.7 (3.2)	7.5 (2.5)	.13
Dilatation in penultimate hour, median (IQR), cm	8 (5-10)	8 (4-10)	.13
Fully dilated or dilated 9 cm in penultimate hour	16 (45.7)	49 (46.7)	.92
Cord blood gas results			
pH, mean (SD)^a^	6.97 (0.17)	NA	NA
Base deficit, mean (SD), mmol/L^b^	13.7 (9.47)	NA	NA
Lactate, mean (SD), mg/dL^c^	100.9 (35.1)	NA	NA

Abbreviations: BMI, body mass index (calculated as weight in kilograms divided by height in meters squared); IQR, interquartile range; NA, not applicable; VBAC, vaginal birth after cesarean.

SI conversion: To convert lactate to millimoles per liter, multiply by 0.111.

^a^ Data available for 31 infants.

^b^ Data available for 22 infants.

^c^ Data available for 14 infants.

Intra-assessor agreement ranged from 63% to 93% for identifying the CTG result as abnormal and 70% to 93% for planning immediate action. Interassessor agreement for immediate action is shown with percentage agreement in the upper triangle of the table and the κ statistic in the lower triangle (Table 3).

**Table 3.** Intra-assessor Agreement for 10 Scorers on 30 Consecutive Reviews for Agreement on Opinion and Recommended Immediate Action and Interassessor Agreement on Immediate Action From 140 Reviews

Scorer	Intra-assessor Agreement, %		Scorer^a^									
	Opinion	Action	1	2	3	4	5	6	7	8	9	10
1	70	70		62%	63%	64%	68%	64%	68%	68%	63%	62%
2	77	80	0.10		85%	84%	80%	75%	81%	72%	85%	90%
3	67	93	0.12	0.24		83%	80%	75%	85%	73%	86%	91%
4	83	90	0.17	0.36	0.35		84%	73%	81%	72%	85%	86%
5	67	90	0.28	0.26	0.29	0.49		78%	78%	72%	83%	81%
6	63	87	0.23	0.29	0.28	0.30	0.43		69%	75%	81%	75%
7	80	83	0.25	0.08	0.28	0.27	0.23	0.14		70%	78%	84%
8	77	87	0.31	0.22	0.25	0.28	0.30	0.41	0.15		76%	75%
9	90	90	0.15	0.41	0.44	0.49	0.48	0.51	0.15	0.38		85%
10	93	93	0.09	0.41	0.47	0.38	0.27	0.27	0.13	0.28	0.36	

^a^ Interassessor agreement scores are presented as percentages and κ statistics.

Table 4 shows the proportions of cases and controls assessed as having an abnormal CTG reading and the cases for which immediate action was proposed by all 10 assessors. Therefore, among all assessors, mean (range) sensitivity for the detection of NE among cases was 75% (63%-91%), and the mean (range) sensitivity for recommending immediate action was 41% (23%-57%). Mean (range) specificity (ie, proportion of control CTGs assessed as not abnormal and not requiring immediate action) was 67% (53%-77%) for detection and 87% (65%-99%) for no action.

**Table 4.** Sensitivity and Specificity for Cardiotocography Readings and Action Plans by Case Status

Scorer	%			
	Cases	Controls	Cases	Controls
	Abnormal	Not Abnormal	Immediate Action^a^	No Immediate Action
1^b^	71	76	54	65
2	69	71	29	95
3	80	62	25	96
4	63	65	43	90
5	80	57	49	88
6^b^	77	69	54	76
7^b^	66	72	23	91
8^b^	80	63	57	78
9	91	53	43	89
10	71	77	29	99
Mean (range)^c^	75 (63-91)	67 (53-77)	41 (23-57)	87 (65-99)
Mean (range)^d^	76 (63-91)	71 (53-77)	36 (25-49)	93 (88-99)

^a^ Immediate action defined as fetal blood sample or immediate delivery.

^b^ Assessor had poor interassessor agreement (ie, <80%) for immediate action. These assessors were excluded from the sensitivity analysis.

^c^ Includes all assessors.

^d^ Mean of 6 assessors with good interassessor agreement.

A sensitivity analysis removing assessors 1, 6, 7, and 8 (ie, those with <80% agreement in Table 3) is reported in Table 4 and shows the proportion of cases and controls assessed as having an abnormal CTG reading and the proportion for which immediate action was proposed by the 6 reliable assessors. The mean (range) sensitivity for detection of NE and for recommending immediate action among the 6 reliable assessors was 76% (63%-91%) and 36% (25%-49%), respectively. Mean (range) specificity (ie, proportion of cases in which control CTGs were assessed as not abnormal and not requiring immediate action) was 71% (53%-77%) for detection and 93% (88%-99%) for action.

## Discussion

The aim of this study was to determine whether experienced clinicians could detect abnormal CTG readings during the penultimate hour before delivery and recommend immediate action (and, therefore, earlier birth) for infants diagnosed with moderate to severe NE. We found that clinicians were able to detect three-quarters of CTGs for infants diagnosed with NE as abnormal at least 1 hour before birth and to recommend expedited birth in nearly half of cases.

This study highlights the variability in interpretation of CTG readings, even among experienced clinicians who had recently completed a course in fetal surveillance education. It also raises concerns about the lack of competency assessment following fetal surveillance education. Pretesting and posttesting of competence has been shown to enhance the learning experience.^21^ However, most assessors only agreed with themselves 70% to 80% of the time. This point also highlights the lack of a criterion standard for interpretation of CTGs. Perhaps the only criterion standard is the correct assessment of a CTG and appropriate action plan when birth asphyxia was present and resulted in NE.

The CTG assessment tool used in this study was recommended by RANZCOG at the time the study was undertaken; however, it has since undergone minor revisions, including removing the suspicious category. The tool is based on the National Institute of Clinical Excellence guidelines and elements of the RANZCOG guidance on CTG interpretation (Table 1).^11,12^ The specific aim of the RANZCOG guideline is to reduce adverse perinatal outcomes associated with inappropriate or inadequate intrapartum fetal surveillance. A 2017 study comparing the agreement and accuracy of the International Federation of Gynecology and Obstetrics, American College of Obstetricians and Gynecologists, and National Institute of Clinical Excellence CTG interpretation guidelines^22^ reported that the International Federation of Gynecology and Obstetrics and National Institute of Clinical Excellence guidelines showed a trend toward higher sensitivities in the prediction of newborn acidemia (89% and 97%, respectively) than the American College of Obstetricians and Gynecologists guideline (32%), but the latter achieved a significantly higher specificity (95%). The reported sensitivities were higher than our study (75%); however, in the previous study, only 5% infants were born with acidemia and there were no cases of NE.^22^ The sensitivity of 75% is not surprising given that randomized studies of intrapartum CTGs have consistently failed to report benefit (with the exception of a reduction of neonatal seizures) and the addition of fetal blood sampling, fetal electroencephalography, fetal electrocardiogram ST segment analysis, fetal pulse oximetry, and computerized CTGs have not delivered hoped-for improvements in outcomes.^13,15,23^ To summarize, even with these additional features, CTGs have not been found to improve outcomes associated with birth asphyxia. Additionally, they have been associated with increases in operative vaginal delivery and cesarean delivery. In this study, experienced clinicians who had completed a fetal surveillance education program within 2 years detected 3 of 4 infants with asphyxia leading to NE by CTG and created a plan to expedite delivery in nearly half of cases. These findings support the further investment in education in the interpretation of CTGs.^24^ We acknowledge that the tool is not perfect, but a lack of education will exaggerate any tool limitations. We propose that fetal surveillance education be mandatory and include competence testing.^25,26^ Currently, fetal surveillance education is not mandatory in any country.^26^ The lack of mandatory education and having a pass or fail test at the end of the education program are 2 areas on which further research could be focused. The concept of double reading each CTG is also an area for further study.

### Strengths and Limitations

A strength of our study was that we have minimized hindsight bias, in which CTGs from infants diagnosed with NE are assessed retrospectively and unmasked.^9^ Knowledge of the perinatal outcome influences the assessment of the CTG.^27,28^ For example, in a study of 42 fetal tracings reviewed by 123 health care professionals in which the perinatal outcome was initially withheld and later disclosed, hindsight resulted in a more pessimistic assessment.^28^ Our study design avoided unmasked assessment by not revealing the perinatal outcome.^29,30^ To our knowledge, only 1 other study has used a similar methodology, embedded cases with normal CTGs, and masked assessors to the perinatal outcome.^15^ That study of 107 cases and 107 controls concluded that CTGs had low predictive value in the identification of fetal hypoxia and “that EFM [electronic fetal monitoring] is not a precise tool in the identification of metabolic acidosis or HIE [hypoxic-ischemic encephalopathy].”^15^ The previous study only assessed the last hour or 30 minutes of CTG before delivery, did not report on expediting delivery, and included a population at high risk, given that the average gestational age of cases and controls was less than 37 weeks and the neonatal mortality rate among the controls was 19 of 1000 births.

A further strength of our study was the assessment of intra-assessor and interassessor agreement. We asked all assessors to repeat the assessment on 30 CTGs 1 year later. There was moderate intra-assessor agreement for both CTG analysis and for recommended actions, although this was higher for actions than for CTG assessment (ie, all but 1 assessor scored more than 80%). However, the interassessor agreement was poor for 4 of 10 assessors, and the results of those 4 assessors were excluded in a sensitivity analysis. Other studies have reported even lower proportions of interassessor agreement.^31,32^ Our rationale for excluding assessors with low interassessor agreement in a sensitivity analysis was a priori concerns that completion of fetal surveillance education in itself was not a marker of competence.

This study has limitations. First, only the penultimate hour of the CTG was considered. We took this approach as current RANZCOG guidelines recommend 1 hour of assessment and do not recommend taking a holistic judgement of the whole CTG. Second, the survey was an artificial setting with no real-world pressure on the clinicians. Third, we cannot fully rule out the possibility of an antenatal cause of NE even though previous in-depth reviews of the cases did not find other causes.^9^ There is considerable debate about the causes of NE in the absence of an acute peripartum event.^33,34^ Although it is possible that some of the cases included in this study were not caused by acute peripartum asphyxia because of the criteria used for inclusion, the result of this limitation would be to negate any potential for CTG to improve outcomes during the penultimate hour before birth. Fourth, we acknowledge that there may be concerns about the inclusion criteria we used for the cases and that this might have resulted in including cases in which peripartum hypoxia was not the cause of NE. The pH threshold was chosen for the original case review study and supported by the American College of Obstetricians and Gynecologists and American Academy of Pediatrics 2014 statement, which reads, “if the cord arterial gas pH levels are above 7.20, it is unlikely that intrapartum hypoxia played a role in causing neonatal encephalopathy.”^8^ Data from the cases suggest that most case infants had very abnormal blood gases along with moderate or severe NE. We did allow inclusion based on venous samples, supported by the literature,^35^ although only 1 case relied on the venous sample as an inclusion criteria.

## Conclusions

This study found that clinicians were able to detect abnormal CTG readings for three-quarters of infants diagnosed with NE 1 hour before birth and to recommend expedited birth in nearly half of cases. It is clear that some infants who were born with birth asphyxia and developed NE went undetected with and without the use of CTGs. We suggest that only those clinicians who attain a predefined level of competence in CTG interpretation should be able to provide secondary care in labor. The use of the CTG is firmly embedded in maternity practice. Our study highlights the need for further investment in and exploration of new approaches to fetal surveillance in labor. If this is as good as it gets, then we will continue to see rising intervention rates in women with no fetal hypoxia while continuing to fail some infants at risk.

## Notes

Supplement. **eTable 1.** Criteria for Inclusion Among Study Cases **eTable 2. **Action Options for Clinical Care in Response to CTG Findings
