# Supplementary material for: Clinician Identification of Birth Asphyxia Using Intrapartum Cardiotocography Among Neonates With and Without Encephalopathy in New Zealand
Source: JAMA Netw Open. 2020 Feb 19;3(2):e1921363. doi: 10.1001/jamanetworkopen.2019.21363 (PMC12549159; doi:10.1001/jamanetworkopen.2019.21363)
Supplement: Supplement. — eTable 1. Criteria for Inclusion Among Study Cases eTable 2. Action Options for Clinical Care in Response to CTG Findings [file jamanetwopen-e1921363-s001.pdf]

Supplementary Online Content

Farquhar CM, Armstrong S, Masson V, Thompson JMD, Sadler L. Clinician identification of birth asphyxia using intrapartum cardiotocography among neonates with and without encephalopathy in New Zealand. *JAMA Netw Open*. 2020;3(2):e1921363. doi:10.1001/jamanetworkopen.2019.21363

eTable 1. Criteria for Inclusion Among Study Cases
eTable 2. Action Options for Clinical Care in Response to CTG Findings

This supplementary material has been provided by the authors to give readers additional information about their work.

© 2020 Farquhar CM et al. *JAMA Network Open.*

eTable 1. Criteria for Inclusion Among Study Cases

Cord arterial and venous or arterial* blood gas results, one minute Apgar scores, and number of inclusion criteria met

pH		Base Deficit(mmol/L)		Lactate (mmol/l)		Apgar score: 1 Minute	Criteria met
Arterial	Venous	Arterial	Venous	Arterial	Venous		
**6.57**	**6.71**	**-30**	**-26**			**1**	3
**6.57**		**-28**				**2**	3
**6.73**	**6.73**		**-27**			**1**	3
**6.74**	**6.74**					**7**	2
**6.8**	**6.88**		**-29**			**1**	3
**6.8**	**7.2**	**-23**		**11.3**	**6**	**0**	4
**6.82**	**6.82**		**-16.3**		**22**	**0**	4
**6.85**	**6.97**	**-17.6**	**-14.1**			**5**	3
**6.87**	**6.87**		**-13.6**			**3**	2
**6.9**	7.3	**-19**	**-10**			**4**	3
**6.92**	**7.12**	**-16**	**-14**	**13.1**	**7.6**	**4**	4
**6.94**	**6.94**		**-15**		**10.2**	**4**	3
**6.95**		**-18**		**9**		**3**	4
**6.96**	**7.03**	**-16**	**-13**			**1**	3
**6.97**	**7.04**	**-18**	**-15**			**5**	3
**6.97**	7.22	**-15**	-8	**11.4**		**2**	4
**7.04**		**-11**				**1**	3
**7.05**		**-11.1**		**7**		**0**	4
**7.06**	**7.06**		**-15**			**1**	3
**7.07**						**6**	2
**7.08**		-9				**6**	2
**7.11**	**7.2**	-9.8	-9.8	**11.5**	**9.7**	**2**	3
**7.13**		10		**6.9**		**2**	3
**7.14**	**7.14**	-9.1	-9.1			**4**	2
**7.14**	**7.2**	**-11.5**	-9	**8.6**	**6.7**	**4**	4
**7.15**		-1.5		**8**		**1**	3
**7.16**		**-10.1**		**13.7**		**2**	4
**7.17**		**-19**		**14.3**		**0**	4
**7.2**	7.27	**-10**	**-10**			**1**	3
**7.2**	7.3	-4	-3			**0**	2
7.3	**6.91**	5		3.1	**10.2**	**0**	3
No blood taken						**7**	1
No blood taken						**1**	1
No blood taken						**5**	1
No blood taken						**2**	1

*Arterial samples collected within one hour of birth
Bolded results indicate where inclusion criteria were met

© 2020 Farquhar CM et al. *JAMA Network Open.*

eTable 2. Action Options for Clinical Care in Response to CTG Findings

1	Discontinue CTG and commence intermittent auscultation
2	Continue to monitor with continuous CTG and review CTG after short interval +/- other measures as indicated e.g. re-position woman, intravenous fluids, stop syntocinon, administer intravenous antibiotics
3	Perform fetal blood sample and clinical plan dependent on result
4	Category one (immediate) delivery either by caesarean section or instrumental delivery dependent on your clinical findings
5	Category two (urgent) delivery either by caesarean section or instrumental delivery dependent on your clinical findings

© 2020 Farquhar CM et al. *JAMA Network Open.*
